# Nuclease-Assisted Suppression of Human DNA Background in Sepsis

**DOI:** 10.1371/journal.pone.0103610

**Published:** 2014-07-30

**Authors:** Yajing Song, Christian G. Giske, Patrik Gille-Johnson, Olof Emanuelsson, Joakim Lundeberg, Peter Gyarmati

**Affiliations:** 1 Royal Institute of Technology, Science for Life Laboratory, Stockholm, Sweden; 2 Karolinska Institutet, Department of Microbiology, Tumor and Cell Biology, Stockholm, Sweden; 3 Karolinska University Hospital, Department of Clinical Microbiology, Stockholm, Sweden; 4 Division of Infectious Diseases, Department of Medicine, Karolinska Institutet, Stockholm, Sweden; University of California Merced, United States of America

## Abstract

Sepsis is a severe medical condition characterized by a systemic inflammatory response of the body caused by pathogenic microorganisms in the bloodstream. Blood or plasma is typically used for diagnosis, both containing large amount of human DNA, greatly exceeding the DNA of microbial origin. In order to enrich bacterial DNA, we applied the C_0_t effect to reduce human DNA background: a model system was set up with human and *Escherichia coli (E. coli)* DNA to mimic the conditions of bloodstream infections; and this system was adapted to plasma and blood samples from septic patients. As a consequence of the C_0_t effect, abundant DNA hybridizes faster than rare DNA. Following denaturation and re-hybridization, the amount of abundant DNA can be decreased with the application of double strand specific nucleases, leaving the non-hybridized rare DNA intact. Our experiments show that human DNA concentration can be reduced approximately 100,000-fold without affecting the *E. coli* DNA concentration in a model system with similarly sized amplicons. With clinical samples, the human DNA background was decreased 100-fold, as bacterial genomes are approximately 1,000-fold smaller compared to the human genome. According to our results, background suppression can be a valuable tool to enrich rare DNA in clinical samples where a high amount of background DNA can be found.

## Introduction

DNA diagnostic systems, such as PCR, microarray and sequencing, require high sensitivity and specificity for accurate detection. However, huge amount of background DNA can hamper these properties, leaving rare DNA undetected; while partial removal of human DNA background has been shown to be beneficial for diagnostic systems [1]. In order to reduce abundant DNA in a sample, the C_0_t effect [2] can be utilized: following denaturation, abundant DNA hybridizes faster than rare DNA, thus with the application of nucleases specific to double stranded DNA, the amount of abundant DNA can be decreased.

The C_0_t-effect and nucleases specific for double stranded DNA can therefore improve detection of rare targets [3], as nucleases degrade the hybridized dsDNA but leave the non-hybridized (therefore single stranded) rare DNA intact. In case of blood or plasma samples, DNA has to be fragmented before this treatment in order to make human and bacterial DNA sizes uniform; also, short DNA fragments (200–300 bp) hybridize quicker than several kilobase long segments. Here we describe a quantification of the C_0_t effect and a clinical application of this approach by demonstrating reduced human background DNA on a model system containing amplicons and on clinical samples (plasma and blood) from septic patients.

Detection of rare DNA is crucial in bloodstream infections, such as sepsis, because viable pathogens can be present at low copies, even as low as one copy/ml [4]–[6] and the amount of human DNA can exceed the amount of pathogen DNA over a million-fold [7]. Sepsis is a life threatening medical condition and the most common source of death in critically ill patients, with over 200,000 cases per year in the United States [8], [9]. Early and precise diagnosis is crucial in sepsis, as survival rate decreases quickly by time [10] and the given treatment should be specific to avoid antibiotic misuse. The current diagnostic gold standard of blood stream infections (BSI) involves identification of the causative pathogen through blood cultures, a time- consuming process which may take several days. The delayed diagnosis is compensated for by empirical treatment with broad-spectrum antimicrobials to cover a range of possible pathogens, a strategy which may contribute to the escalation of antimicrobial resistance. Consequently, there is an urgent need for more rapid diagnostic tools in order to achieve better tailored therapies [6], [11].

Real-time PCR could be an ideal method for diagnosing sepsis due to its high sensitivity and specificity, rapid turnaround time, and broad dynamic range [10], [12], as pathogen amount can vary between 1 fg-100 ng per 1 ml blood [4]. However, detecting minute amount of pathogen DNA can be challenging for PCR systems [4], [7], as inhibitors and the huge excess of human DNA found in blood can suppress PCR efficiency [7], [13], [14]. Commercially available detection systems did not eliminate these problems either [10], [15]–[17].

Blood is commonly used as a testing material for sepsis but plasma can be used for pathogen detection as well [10], [12], as it reduces the overall DNA amount. One hundred µl blood can contain >1 µg DNA, while 100 µl plasma contains ∼100 ng DNA, but it can vary depending on the disease state [18]. Pathogen concentration can be as low as 1 copy/ml which would mean ∼5 fg DNA for *E. coli*, the most common Gram-negative pathogen in sepsis cases [5]. Our goal in this study was to demonstrate and quantify nuclease-assisted background suppression on a model system using amplicons; and applying this method on clinical samples from septic patients. With the presented nuclease-assisted suppression of human DNA in septic samples, the human DNA has been decreased 100-fold in order to improve the detection of very low pathogen concentrations, which commonly occur in bloodstream infections, such as sepsis [1], [4], [5].

## Materials and Methods

### Primers

Primers were designed to specifically detect and quantify human and *E. coli* DNA. *E. coli* primers target the 1-deoxyxylulose-5-phosphate synthase gene (Fwd: 5′-GGCGAGAAACTGGCGATCCTTA-3′, rev: 5′-CGCTTCATCAAGCGGTTTCACA-3′), a gene which shows high level of conservation in *E. coli* [19]. Human primers target the β-actin gene (Fwd: 5′- CCCTTCCCCCTTTTTTGTC-3′, rev: 5′-CAACTGGTCTCAAGTCAGTG-3′). Primers were cross-checked for mispriming and secondary structures. Oligonucleotides were ordered from Cybergene AB (Stockholm, Sweden) and MWG (Ebersberg, Germany).

### DNA samples

Amplicons were generated in a classical PCR reaction, containing 1 U Platinum Taq DNA Polymerase (Invitrogen, Carlsbad, CA, USA), 1× Platinum Taq PCR buffer (Invitrogen), 0.2 mM dNTPs, 1.5 mM MgCl_2_, 200 nM each of a forward primer and reverse primer, and 1 ul extracted sample DNA in 50 ul PCR solution. PCR reactions were performed at 94°C for 5 min, and cycled 45 times through 94°C for 30 s, 58–61°C for 40 s, and 72°C for 60 s, and elongation step of 72°C for 10 min was processed following the cyclic amplification, then size was controlled on a 2% agarose gel and with a 2100 Bioanalyzer, using Agilent DNA 1000 chips. Amplicons were purified by using Qiagen QIAquick Gel Extraction Kit (Qiagen, Venlo, Netherlands).

Clinical samples were obtained from the Karolinska University Hospital from patients having ≥2 SIRS (systemic inflammatory response syndrome) criteria [20] and were qPCR positive for *E. coli* based on the amplification and melting curves. Filtered human plasma was used as negative control (Sigma, St. Louis, MO, USA). DNA has been extracted using the Qiagen DNeasy Blood&Tissue Kit.

**Figure 1:**
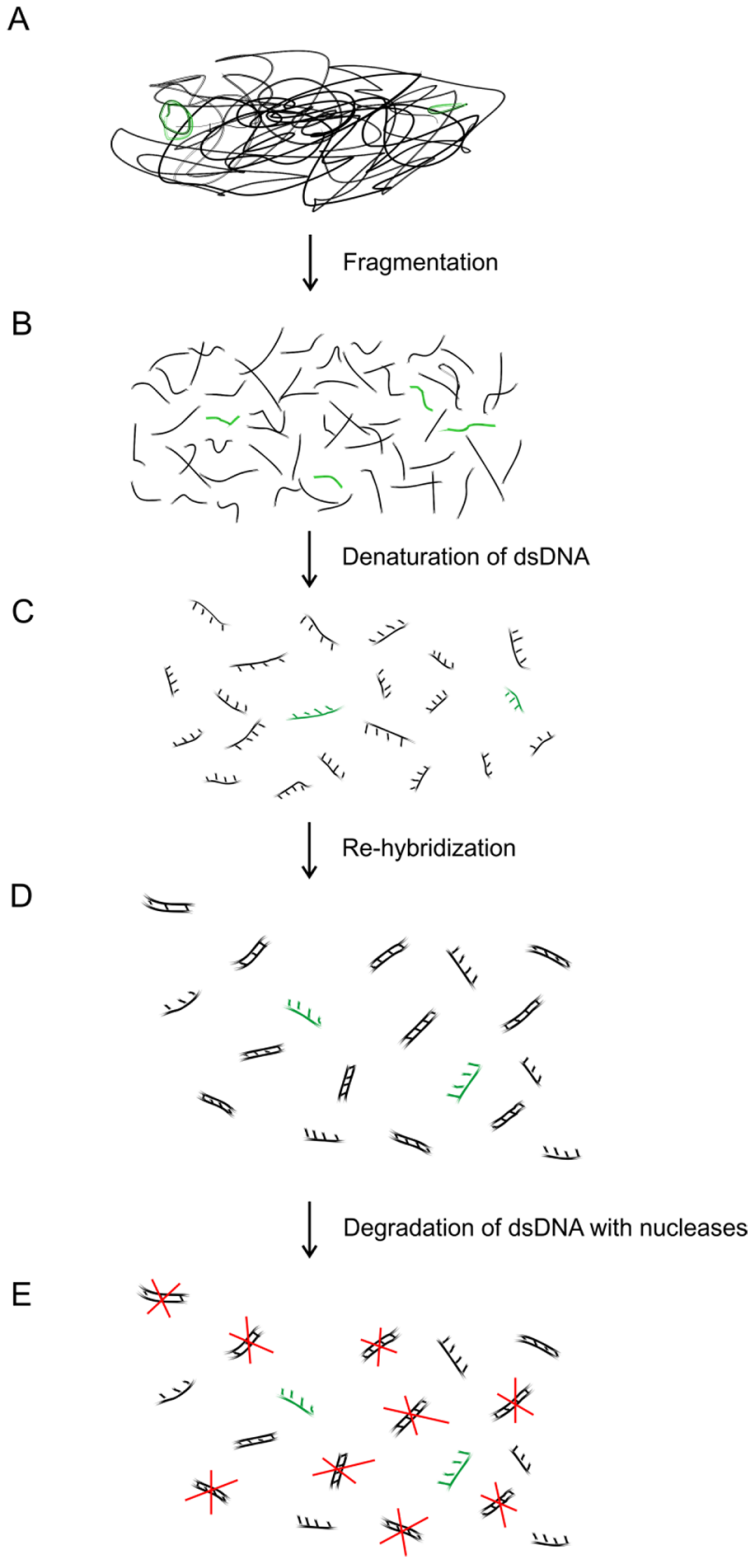
Schematic representation of the presented background suppression method. In septic blood, the amount of human DNA (black) exceeds pathogen DNA (green) amount (A). The extracted DNA is fragmented, denatured and re-hybridized, then dsDNA is degraded with nucleases (red) specific to double stranded DNA. Since rare DNA re-hybridizes slower, mostly abundant DNA will be degraded.

Whole blood samples were processed with the MolYsis Complete5 kit (Molzym Life Science, Bremen, Germany) as described by the manufacturer, followed by fragmentation, rehybridization and nuclease treatment (Figure 1). The study was approved by the Regional Ethical Review Board. Informed written consent was obtained from all participants.

### Rehybridization and nuclease treatment

A mixture of human and *E.coli* amplicons (ratio of 10^8^∶ 1) was denatured at 98°C for 2 min, and rehybridized at 68°C for 5 hours. DSN (duplex-specific nuclease; Evrogen, Moscow, Russia) treatment was carried out as 30 min with 0.25 U DSN at 68°C in 1x DSN buffer.

Clinical samples were processed to chemical fragmentation using the iron-EDTA method: 20 mM ammonium iron(II) sulfate hexahydrate and 40 mM EDTA solutions were pre-mixed, then 0.1 M dithiothreitol and 1% hydrogen peroxide have been added and homogenized through pipetting (reagents were obtained from Sigma, St. Louis, MO, USA). Finally, the mixture was precipitated with 3 volumes isopropanol [21]. Then double stranded DNA was denatured at 98°C for 3 min, and re-hybridized at 55°C for 30 min using a thermocycler. One U of DSN and 1x DSN buffer were added to the reaction mixture in a total volume of 10 µl, and then incubated at 61°C for 30 min, followed by 1 U of BAL 31 (New England Biolabs, Ipswich, MA, USA) at 30°C for 10 min (Figure 1). Buffer exchange was carried out with the Zymo DNA Clean and Concentrator Kit (Zymo Res. Corp., Irvine, CA, USA).

### Real-time PCR

Reactions containing 1x EvaGreen SYBR Green supermix (Bio-Rad Laboratories, Hercules, CA, USA), 400 nM of human β-actin primers, 200 nM *E. coli* primers and 5 µl DNA in a 20 µl mixture. Thermal conditions were: 95°C for 3 min, then cycled 50 times at 95°C for 30 sec, 61°C for 30 sec and 72°C for 1 min. Melting curves were generated with 0.5°C increments between 61–95°C. Amplicon and plasma samples have been run on a Bio-Rad CFX96 instrument, while a Rotor-Gene 3000 instrument was used for blood samples. P-values were determined using Student’s *t*-test with significance level set to 5%.

### Generation of standard curves

**Figure 2:**
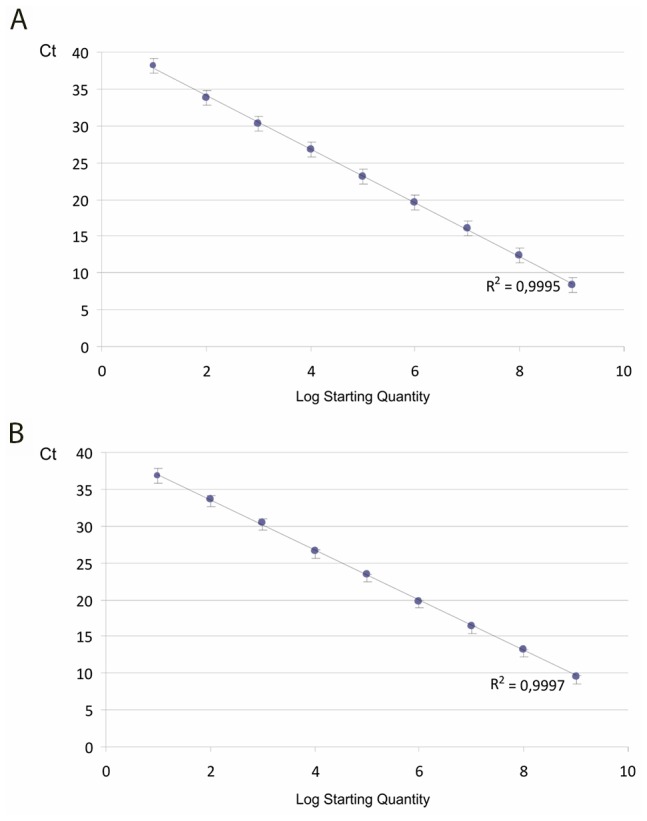
Standard curves and nuclease effect using amplicons to model excess human DNA. Human β-actin (a) and *E. coli* (b) primers provided the same R2 (>0.99) value and sensitivity (1–10 copies as the lower limit of detection) on amplicons in triplicate trials. ΔCt was 3.2 for human, 3.37 for *E. coli*. Error bars represent standard deviation.

Ten-fold dilutions of human β-actin and *E. coli* were prepared from 10^0^–10^9^ copies (Figure 2). Standard curves were generated using the Bio-Rad CFX Manager software (v 1.6.541).

## Results

**Figure 3:**
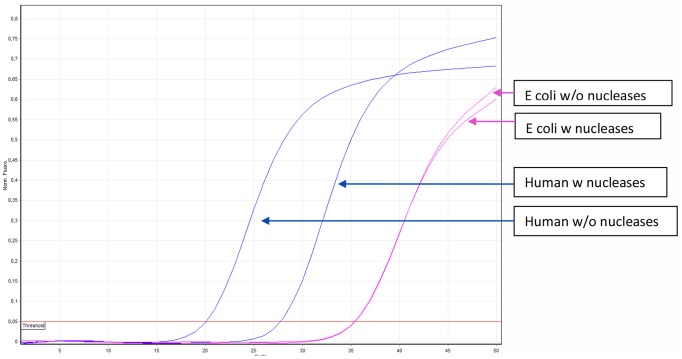
Representative amplification curves of 10 experiments show background suppression on a septic blood sample, with blue lines representing the human DNA (β-actin), and pink lines show *E. coli* DNA amount. While the amount of human DNA has degraded, the *E. coli* DNA amount did not change.

The efficiency of background suppression of human DNA was evaluated in three different ways: 1) with amplicons resulting from PCRs targeting β-actin and *E. coli* in order to optimize the method, 2) with clinical plasma, and 3) blood samples from septic patients to prove the usability of the method (Figures S1–S2, Figure 3). In addition to the amplification curves, melting peaks specific for either β-actin or *E. coli* amplicons were used to identify positive signals (Figure S2b).

### Standard curves

Standard curves were used to compare the efficiency of human and *E. coli* PCR systems, with a serial dilution of amplicons. Both PCR systems showed a high linear relationship, reflected by the R^2^ values (>0.99) and high reproducibility with triplicate trials (Figure 2).

### Background suppression on amplicons

The detection of microbial DNA in bloodstream infections usually suffers from high human background DNA, as the amount of pathogens can be as low as one or few copies in 1 ml of blood, equal to approximately 1–100 fg of DNA, with micrograms of human DNA. Modeling this ratio, human β-actin amplicons and amplicons from the *E. coli* 1-deoxyxylulose-5-phosphate synthase gene were mixed, reflecting a 10^8^–fold excess of human DNA copies. Application of DSN decreased the human DNA background by 100,000-fold (Figure S1) estimated by the ΔΔC_t_ method [22], [23]. In order to avoid stochastic effects of low-copy detection on concentration estimates [24], triplicate experiments were performed.

### Plasma samples from septic patients

The nuclease treatment of clinical samples were optimized by human and *E. coli* genomic DNA in sterile plasma. Five plasma samples from septic patients were collected and all samples were positive for *E. coli* in qPCR. Clinical samples contained 1.3 ng/µl DNA±0.1 (mean ± SD). The human DNA exceeded pathogen DNA approximately 500-fold (8.8 Ct average), indicating a ∼500,000-fold excess in mass. After the application of nuclease treatment, the β-actin amplification curve was suppressed with an average 5.5±1.2 cycles (mean ± SD), while the *E. coli* amplification curve was suppressed with 0.3±0.5 cycles (p<0.05, Figure S2a), suggesting an approximately 45-fold enrichment in pathogen DNA [22], [23].

### Whole blood samples from septic patients

Ten blood samples from septic patients were collected at the Karolinska University Hospital. The samples were processed to MolYsis treatment, resulting in an average DNA concentration of 14.4 ng/ul. After nuclease treatments, the β-actin DNA was suppressed with an average 7.0±1 cycles (mean ± SD), while *E. coli* DNA was suppressed with 0.45±0.5 cycles (p<0.05), suggesting an >100-fold enrichment in pathogen DNA (Figure 3).

## Discussion

Sensitivity and specificity are great challenges for any DNA detection or analysis systems in the presence of a high background noise [1]. Samples from bloodstream infections usually contain host DNA in a million-or billion-fold excess compared to pathogen DNA, which can make molecular biological detection systems, such as PCR, less sensitive than blood culture [11]. Background suppression can reduce the amount of human DNA, making PCR more sensitive because of less mispriming and more efficient amplification [1], [7], therefore enhancing the chance of detection. Microarray and sequencing studies also benefit from enrichment of rare DNA [25]–[27].

Suppression with nucleases has been shown to be efficient to reach ultra-sensitive diagnosis [8], [28], [29]. In the present work, a nuclease assisted background suppression method has been utilized and shown to be effective in amplicons, where the copies of human and pathogen DNA reflected the abundance of human DNA. In this model system, a 100,000-fold reduction of human DNA was achieved while the pathogen amount remained unchanged. The efficiency of nuclease treatments was also demonstrated in clinical plasma and blood samples from septic patients where human DNA amount exceeded pathogen DNA.

Extraction from whole blood samples might provide higher amount of pathogen DNAs but their overall concentration is lower than in plasma; also, blood can contain PCR inhibitors [12], [29], which can cause low sensitivity for PCR-based detection [1], [11].

Contrary to lysis-based background suppression methods [7], [14], the method described here can be applied to both blood and plasma samples and use only minute amount of enzymes. Also, as it has been shown, the presented treatment affected bacterial DNA to a minimal degree (Figure 3, Figures S1–S2a), while degradation of pathogen DNA has been observed in other systems [14]. This method is aspecific to the type or origin of abundant DNA, therefore it does not restrict the downstream diagnostic methods to bacteria as seen in commercial systems [1], [10], but might promote the detection of other microorganisms such as viruses and fungi. Not only sensitivity could improve by partial removing of the human DNA background [1], but metagenomic studies would also enormously benefit from this assay by reducing the amount human DNA to be sequenced.

Genomic DNAs contain several fold more DNA than amplicons, and the human genome is approximately 1000-fold larger compared to *E. coli*; as a consequence, human DNA background can be suppressed less efficiently compared to amplicons, as the nucleases digest not only the PCR target regions. However, a 100-fold suppression could be demonstrated on whole blood samples. For genomic DNA in clinical samples, two nucleases were used as we found that digestion from inside and outside of the dsDNA strand with the combination of an exonuclease (BAL 31) and an endonuclease (DSN) provided quicker and more efficient degradation of abundant DNA. This assay takes approximately 2 hours to perform, and it requires equipment commonly used in diagnostic laboratories. Therefore, we propose that this assay could be introduced as a preparatory step preceding DNA-based diagnostic applications in clinical diagnostic laboratories.

### Conclusions

Specimens from bloodstream infections usually contain few copies of pathogens but abundant human DNA. In diseases with high mortality rate such as sepsis a prompt, sensitive and accurate diagnosis is of the highest importance in order to provide adequate treatment. The presented method, by decreasing the level of human background DNA, could facilitate ultra-sensitive detection systems to detect pathogens, which otherwise would go unnoticed in cases where a large amount of background DNA is present.

## Supporting Information

Figure S1 **Nuclease treatment reduced human background.** (cyan amplification curve represents untreated β-actin, red-nuclease treated β-actin, brown-untreated *E. coli*, blue-nuclease treated *E. coli*) approximately 100,000-fold, while *E. coli* amount did not change.
(PDF)

Figure S2 **Human β-actin and** ***E. coli*** **amplification curves from plasma samples of septic patients.** With nuclease treatment, human background was suppressed, while pathogen DNA amount did not show any changes (a). Human and *E. coli* amplicons can be distinguished by their melting peaks (b).
(PDF)
