# Supplementary figures and images for: Nuclease-Assisted Suppression of Human DNA Background in Sepsis
Source: PLoS One. 2014 Jul 30;9(7):e103610. doi: 10.1371/journal.pone.0103610 (PMC4116218; doi:10.1371/journal.pone.0103610)

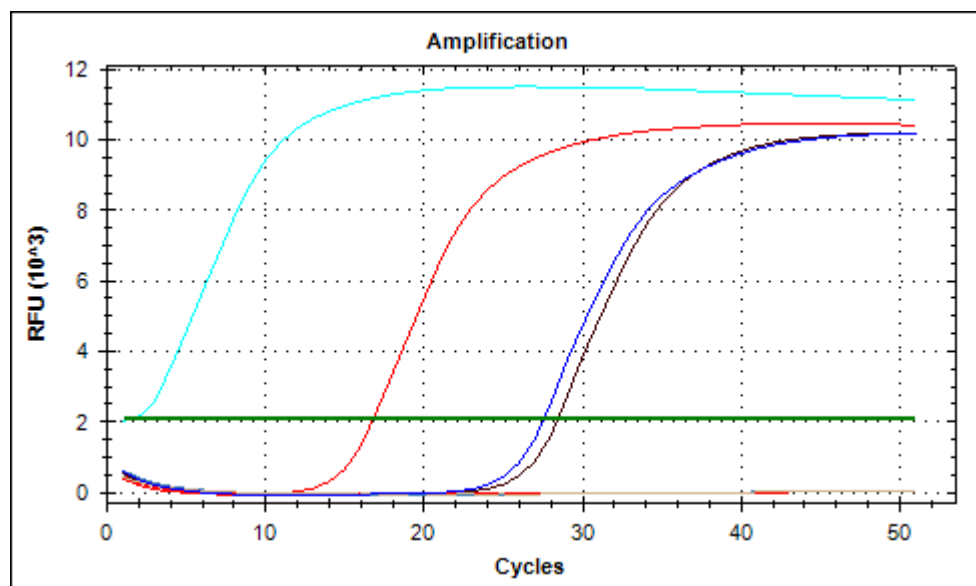

Supplement: Figure S1 — Nuclease treatment reduced human background. (cyan amplification curve represents untreated β-actin, red-nuclease treated β-actin, brown-untreated E. coli, blue-nuclease treated E. coli) approximately 100,000-fold, while E. coli amount did not change. (PDF) [file pone.0103610.s001.pdf]

a)

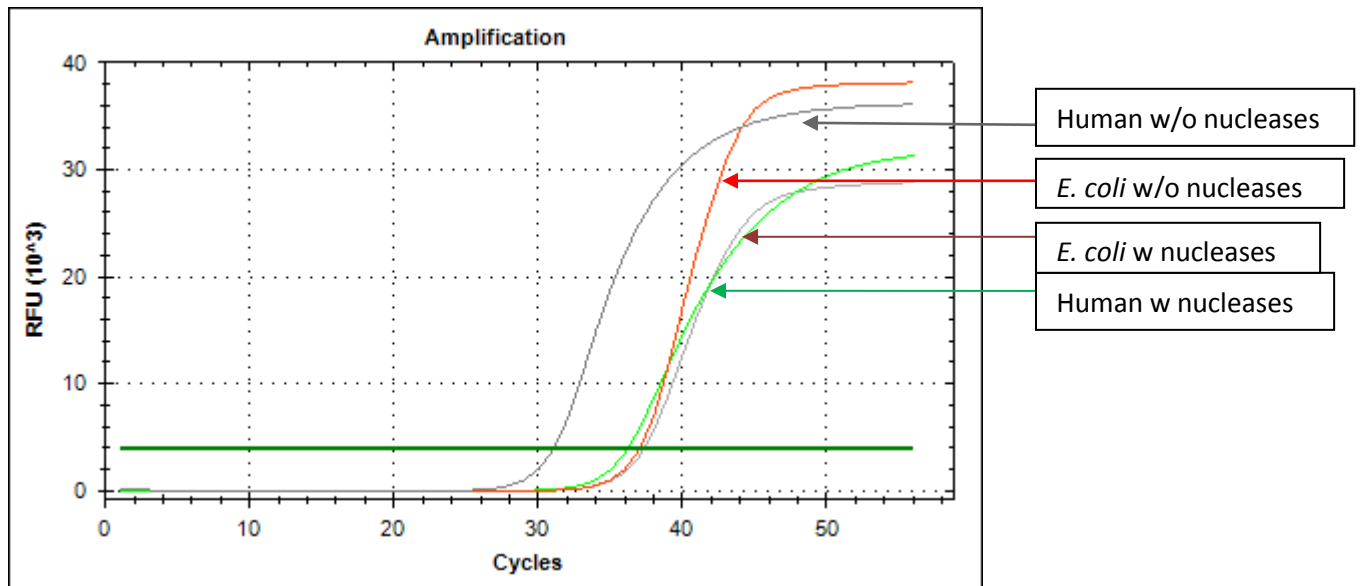

b)

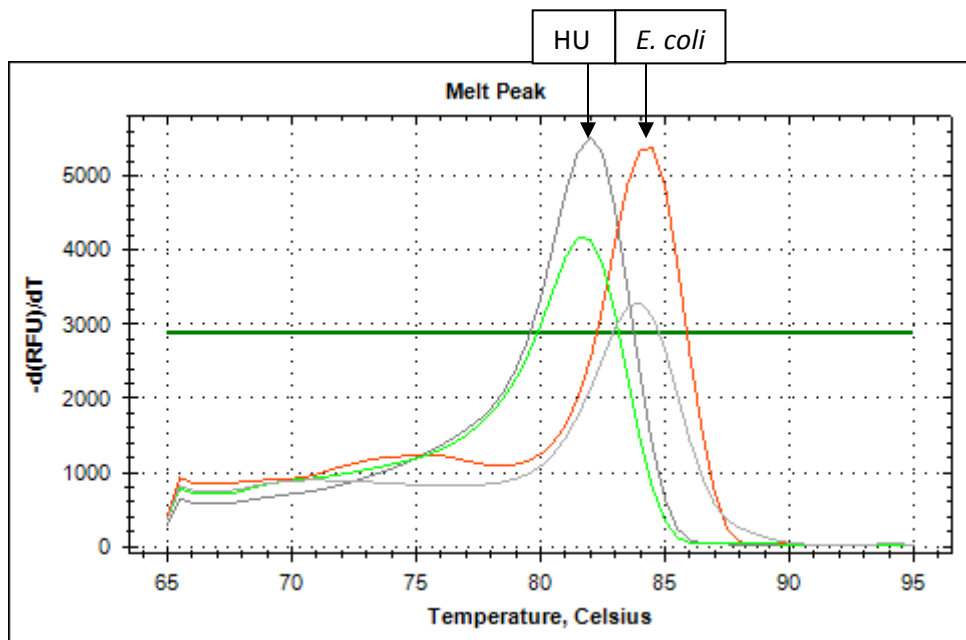

Supplement: Figure S2 — Human β-actin and E. coli amplification curves from plasma samples of septic patients. With nuclease treatment, human background was suppressed, while pathogen DNA amount did not show any changes (a). Human and E. coli amplicons can be distinguished by their melting peaks (b). (PDF) [file pone.0103610.s002.pdf]
